# Role of Patient and Practice Characteristics in Variance of Treatment Quality in Type 2 Diabetes between General Practices

**DOI:** 10.1371/journal.pone.0166012

**Published:** 2016-11-02

**Authors:** Yeon Young Cho, Grigory Sidorenkov, Petra Denig

**Affiliations:** 1 Department of Clinical Pharmacy and Pharmacology, University of Groningen, University Medical Center Groningen, Groningen, the Netherlands; 2 Department of Epidemiology, Graduate School of Public Health, Seoul National University, Seoul, Republic of Korea; Azienda Ospedaliera Universitaria di Perugia, ITALY

## Abstract

**Background:**

Accounting for justifiable variance is important for fair comparisons of treatment quality. The variance between general practices in treatment quality of type 2 diabetes (T2DM) patients may be attributed to the underlying patient population and practice characteristics. The objective of this study is to describe the between practice differences in treatment, and identify patient and practice level characteristics that may explain these differences.

**Methods:**

The data of 24,607 T2DM patients from 183 general practices in the Netherlands were used. Treatment variance was assessed in a cross-sectional manner for: glucose-lowering drugs/metformin, lipid-lowering drugs/statins, blood pressure-lowering drugs/ACE-inhibitor or ARB. Patient characteristics tested were age, gender, diabetes duration, comorbidity, comedication. Practice characteristics were number of T2DM patients, practice type, diabetes assistant available. Multilevel logistic regression was used to examine the between practice variance in treatment and the effect of characteristics on this variance.

**Results:**

Treatment rates varied considerably between practices (IQR 9.5–13.9). The variance at practice level was 7.5% for glucose-lowering drugs, 3.6% for metformin, 3.1% for lipid-lowering drugs, 10.3% for statins, 8.6% for blood pressure-lowering drugs, and 3.9% for ACE-inhibitor/ARB. Patient and practice characteristics explained 19.0%, 7.5%, 20%, 6%, 9.9%, and 13.4% of the variance respectively. Age, multiple chronic drugs, and ≥3 glucose-lowering drugs were the most relevant patient characteristics. Number of T2DM patients per practice was the most relevant practice characteristic.

**Discussion:**

Considerable differences exist between practices in treatment rates. Patients’ age was identified as characteristic that may account for justifiable differences in especially lipid-lowering treatment. Other patient or practice characteristics either do not explain or do not justify the differences.

## Background

Quality assessment frameworks have been introduced in several countries with the goal to ensure appropriate and evidence-based healthcare for patients [1]. Within such frameworks, guideline recommendations on optimal care are linked to performance measures and accountability processes. Performance measures assess to what extent care is delivered according to guideline recommendations. When applying such measures in clinical practice, variation in the performance of healthcare providers and institutions is common. For example, prescribing of preferred drugs by Dutch general practitioners ranged from 35% to 95%, whereas prescribing drug treatment when indicated ranged from 60% to 95% [1]. Differences in performance may be attributed to the underlying patient population as well as to the healthcare provider and the practice organization.

In heterogeneous populations, it is to be expected that healthcare provider’s treatment decisions are influenced by differences in patient characteristics. For example, patients with type 2 diabetes (T2DM) commonly have multiple conditions and risk factors which may require individualized treatment plans. A recent review concluded that patient characteristics, such as marital status and BMI, affect outcome measures for diabetes but no consistent patterns were observed for process measures [2]. Previous research indicates that patient characteristics, such as age [3, 4], gender [5, 6], disease duration [7], comorbidity presence [8], and level of risk factor control [9, 10], can all influence prescribing in this population. This may reflect appropriate care, for example, when different treatment regimens are considered for elderly patients [11, 12], or intensified treatment is prescribed for patients with more progressed disease states [13]. Variance may also be justifiable when there are competing demands [14, 15] or when patients are reluctant or unwilling to take specific or more drugs [16]. In other cases, variance can be non-justifiable, for example, when prescription rates for guideline recommended treatment differ in men and women [5, 6]. Healthcare providers’ personal preferences, knowledge and habits can influence treatment decisions. For example, physicians may differ in their preferences for certain types of treatment, or in their reluctance or keenness to prescribe [10, 17, 18]. In addition, practice characteristics, such as practice type, size, consultation time, and the presence of support staff have been found to influence prescribing behavior [19, 20]. These factors may contribute to non-justifiable variance between practices. Accounting for patient characteristics causing justifiable differences is important for fair between practice comparisons of treatment quality. The aim of this study is to describe the differences in treatment quality of patients with diabetes between general practices, and identify patient and practice level characteristics that may explain these differences.

## Methods

### Study design and setting

We conducted a cross-sectional study to assess treatment quality in 2012 in a large diabetes care group in The Netherlands, which included more than 80% of all general practices in the province of Groningen. Diabetes care groups have been formed after the introduction of bundled payment in 2007 [21]. They are responsible for the organization and provision of diabetes care in accordance with the Dutch clinical practice guidelines.

### Study population and data collection

Data were collected from the GIANTT (Groningen Initiative to Analyze Type 2 Diabetes) database. This regional longitudinal database contains anonymized data extracted from electronic medical records of almost all type 2 diabetes mellitus (T2DM) patients (<1% opted out) managed in general practice [22]. The database includes prescription data, medical history, results from routine laboratory tests and physical examinations. Medical history consists of diagnoses, which are documented in the medical records by means of the International Classification of Primary Care (ICPC) or short text descriptions which are manually coded. From the 189 general practices in GIANTT, 6 were excluded for having incomplete prescription or diagnostic data. Patients diagnosed with T2DM before 1 January 2012 were selected. Patients with incomplete follow-up in 2012, and patients with missing or invalid onset dates for diabetes were excluded.

### Outcomes: treatment measures

Treatment quality was defined as current treatment status, that is, if a patient was treated or not with guideline recommended drug treatment, similar to many of the prescribing measures currently in use in The Netherlands [23]. We focused on whether any drug treatment was prescribed for eligible patients, and whether the first-choice drug class was prescribed for three common risk factors. This includes treatment in T2DM patients with (1) any glucose-lowering drug, (2) any lipid-lowering drug in patients with an additional diagnosis of dyslipidemia, vascular comorbidity or nephropathy, and (3) any blood pressure-lowering drugs in patients with an additional diagnosis of hypertension, vascular comorbidity or nephropathy. In the Netherlands, general practitioners may prescribe any drug to treat a specific risk factor or indication that is allowed on the Dutch market, but some drugs may have restrictions for reimbursement. Any glucose-lowering treatment included: metformin, sulfonylureas, acarbose, thiazolidinediones, dipeptidyl-peptidase-4-inhibitors, insulins, and other blood glucose–lowering drugs (e.g. exenatide, dapagliflozin). Any lipid-lowering treatment included: statins, fibrates, bile acid sequestrants, nicotinic acid and derivatives, and other lipid-modifying drugs (e.g. ezitimibe). Any blood pressure-lowering treatment included: centrally acting antihypertensives, diuretics, β-blockers, calcium channel blockers, and drugs acting on the renin–angiotensin system. Within the above defined patients the first choice drug classes recommended by Dutch guideline for diabetes care [24] comprised of treatment with (1) metformin, (2) statins, and (3) drugs acting on renin-angiotensin-aldosterone system (RAAS-blockers). A patient was considered as being treated when a prescription was recorded within the last 4 months of 2012, taking into account that a prescription can be issued for a maximum period of 3 months in the Netherlands.

### Explanatory variables

To explain the differences in treatment between practices, the following patient level characteristics were included as dichotomous variables: age (≥70 years for glucose lowering treatment, ≥80 years for blood pressure and lipid lowering treatment), gender, duration of diabetes (≥2 years), overweight (ICPC-1 codes: T82, T83), history of cardiovascular comorbidity (ICPC-1 codes: K74-K77, K84, K99.1, left ventricular hypertrophy, coronary artery bypass graft, percutaneous transluminal coronary angioplasty), history of peripheral vascular comorbidity (ICPC-1 codes: K89-K92, peripheral bypass, percutaneous transluminal angioplasty), diabetes complications (ICPC-1 codes: K99.6, F83, N94.2), nephropathy (ICPC-1 codes: U90, U99.1, U99.2, U99.3, dialysis, or kidney transplantation), history of malignancy (ICPC-1 codes: A79, B72-B74, D74-D77, F74.1, H75.1, K72.1, L71.1, N74, R84, R85, S77, T71, U75-U77, X75-X77, Y77, Y78), history of psychological disorders (ICPC-1 codes: P70-P80, P85, P98, P99), treatment with 5 or more other chronic drug classes (ATC codes starting with A, B, C, H, L, M, N, R, excluding antihypertensive, glucose-regulating, and lipid-regulating drugs), treatment with 3 or more glucose lowering drug classes, treatment with 4 or more blood pressure lowering drug classes, and treatment with 2 or more lipid lowering drug classes. The cutoff levels for age were based on the Dutch guideline [24] where less stringent treatment targets are recommended for elderly patients. The cutoff level for diabetes duration was chosen to distinguish patients who were recently diagnosed with T2DM (diabetes duration < 2 years) from those having diabetes for a longer period (≥ 2 years). The cutoff levels for the number of chronic medications and numbers of glucose, blood pressure, and lipid lowering medication were chosen to indicate the burden of being on high numbers of drug classes unrelated to the outcome of interest (e.g. the variable determining treatment with 3 or more glucose lowering drug classes was not used in the models where outcome was defined as treatment with glucose-lowering drugs or metformin). The following general practice level characteristics were included in order to explain potentially non-justifiable differences in treatment between practices: number of diabetes patients per practice, presence of educated diabetes assistant, and practice type (solo or group).

### Statistical analysis

The treatment quality rates were described at practice level as mean percentages with standard deviation, or median percentages with interquartile ranges. Descriptive statistics were also used to describe the distribution of patient characteristics across general practices.

Multilevel logistic regression analysis was conducted for each of the six treatment measures separately (using Stata 14.1, Special Edition) to assess the variance that is attributed to general practice level and the part of this variance that can be explained by patient and practice characteristics. Two level random intercept models were estimated with patients at level 1 nested within general practices at level 2. In these models the probability for the treatment outcome can vary across practices but the effect of the patient characteristics is assumed to be the same (fixed) for all practices.

First, the variance in treatment at practice level was estimated in an empty multilevel model. Second, multilevel univariate analyses were conducted for each patient and practice level explanatory variable. Next, three multivariate models were built using backwards selection for including variables that were potentially associated with the treatment measure (p<0.2); (i) model 1, with patient level characteristics only, (ii) model 2, with general practice characteristics only, (iii) model 3, with patient and practice characteristics together.

The pseudo R^2^ measure was used to estimate what part of the variance at practice level could be explained by including patient and general practice characteristics [25, 26]. For this, the percentages reduction in pseudo R^2^ were calculated for each model compared to the empty model, expressing the part of the practice level variance that can be explained by the included characteristics.

### Ethics statement

In The Netherlands, according to the Code of Conduct for the use of data in Health Research (‘Gedragscode gezondheidsonderzoek’ approved in 2004 by the Dutch College for Protection of Personal Data, taking into account Article 25 of the Dutch Act on the Protection of Personal Data), no ethics committee approval was needed for this research using data from anonymous medical records.

## Result

A cohort of 24,628 patients with T2DM managed in 183 general practices was eligible, after excluding 974 patients for incomplete follow-up and 27 patients for missing or invalid diabetes onset dates. Of the 183 general practices, 90.7% had a diabetes assistant and 45.4% were practices with a single general practitioner (Table 1). The median number of T2DM patients per practice was 122 with a range from 15 to 480 patients. The proportion of patients with comorbidity, hypertension, dyslipidemia and overweight, varied widely across the practices. Among the eligible patients, 75.3% of patients were treated with glucose-lowering drugs, 73.7% with lipid-lowering drugs, and 87.8% with blood pressure-lowering drugs. Considerable differences (IQR 9.5–13.9) were observed in these treatment rates between general practices (Table 2). The between practice variance in the empty multilevel model was 7.5% for glucose-lowering treatment, 3.6% for metformin, 3.1% for lipid-lowering treatment, 10.3% for statins, 8.6% for blood pressure-lowering treatment, and 3.9% for RAAS-blockers (Table 3). The models including all the characteristics (model 3) are presented in Table 4 reflecting the effect sizes of the associations between the included characteristics and treatment outcomes.

**Table 1:** Practice and patients’ characteristics for the study population.

	Number included	%	Median (IQR)	Median (IQR) % or median among practices	Range (Min-Max) among practices
***Practice level***	183				
Diabetes patients per practice			122.0 (77.0)		15–480
Solo practice (%)	83	45.4			
Practice assistant (%)	166	90.7			
***Patient level***	24,628				
Gender, female (%)	12,571	51		50.8 (6.3)	20–65.8
Age (years)	24,628		67.0 (17.0)	67.1 (2.7)	59.5–74.0
Diabetes duration (years)	24,628		5.0 (7.0)	6.2 (1.3)	3.8–9.5
History of cardiovascular comorbidity (%)	5,320	21.6		22.2 (14.9)	0.0–46.5
History of peripheral vascular (%)	2,888	11.7		11.1 (8.9)	0.0–56.0
Diabetes complications (%)	2,380	9.7		8.2 (11.4)	0–30.1
History of malignancy (%)	3,281	13.3		13.7 (11.3)	0.0–53.3
History of psychological disorders (%)	2,500	10.2		8.6 (8.3)	0.0–36.9
Hypertension (%)	12,345	50.1		51.2 (31.7)	1.6–88.2
Dyslipidemia (%)	4,747	19.3		14.6 (19.0)	0.0–79.5
Nephropathy (%)	1,275	5.2		3.3 (6.0)	0.0–30.1
Overweight (%)	11,526	46.8		50.0 (26.6)	3.5–83.1
≥5 chronic drugs (%)	6,916	28.1		26.9 (9.7)	12.4–45.7
≥3 glucose lowering drugs (%)	2,568	10.4		10.5 (6.1)	0.0–20.9
≥2 lipid lowering drugs (%)	753	3.1		2.8 (2.5)	0.0–12.8
≥4 blood pressure lowering drugs (%)	2,939	11.9		11.8 (5.3)	0.0–32.1

**Table 2:** Proportion of patients treated and the between practice differences in treatment rate.

Treatment measure	Patients with treatment indication	Patients receiving treatment	%	Median percentage among practices [IQR]	Range among practices (min–max)
Glucose-lowering drugs	24628	18547	75.3	77.9 (13.9)	39.1–95.7
Metformin	18547	15572	83.9	84.7 (7.5)	60.2–100
Lipid-lowering drugs	10272	7567	73.7	74.3 (13.2)	43.8–100
Statins	7567	7375	97.5	98.6 (3.7)	66.7–100
Blood pressure-lowering drugs	15369	13487	87.8	90.4 (9.5)	55.6–100
RAAS-blockers*	13487	10590	78.5	80.4 (10.2)	45.3–100

* RAAS-blockers: renin-angiotensin-aldosterone system blockers

**Table 3:** Proportion and reduction of variance in treatment attributed to practice level.

	Variance at practice level (%)*				
	Glucose-lowering drugs		Metformin		
	proportion	reduction	proportion	reduction	
Empty model: crude practice level variance	7.5		3.6		
Model 1: including patient characteristics only	7.3	0.2	3.3	0.3	
Model 2: including practice characteristics only	6.4	1.1	3.6	0	
Model 3: including patient and practice characteristics**	6.1	1.4	3.3	0.3	
	Lipid-lowering drugs		Statins		
	proportion	reduction	proportion	reduction	
Empty model: crude practice level variance	3.1		10.3		
Model 1: including patient characteristics only	2.8	0.3	10.2	0.1	
Model 2: including practice characteristics only	2.9	0.2	9.6	0.7	
Model 3: including patient and practice characteristics**	2.5	0.6	9.6	0.7	
	Blood pressure-lowering drugs		RAAS-blockers		
	proportion	reduction	proportion	reduction	
Empty model: crude practice level variance	8.5		3.9		
Model 1: including patient characteristics only	6.7	1.8	3.8	0.1	
Model 2: including practice characteristics only	8.2	0.3	3.7	0.2	
Model 3: including patient and practice characteristics**	6.4	2.1	3.4	0.5	

* Pseudo R^2^ for the two level fixed effect random intercept models

** Model 3 included the variables with the effect size from Table 4

**Table 4:** Effect sizes of the association between the characteristics and treatment outcomes as described in model 3 in the methods (all the selected variables are included into the models).

Model	Treatment with glucose-lowering drugs (n = 24628)	Treatment with metformin (n = 18547)	Treatment with lipid-lowering drugs (n = 10272)	Treatment with statins (n = 7567)	Treatment with blood pressure-lowering drugs (n = 15369)	Treatment with RAAS-blockers (n = 13487)
Age	0.76 (0.71–0.81)	0.55 (0.50–0.60)	0.37 (0.33–0.41)	NA	0.68 (0.60–0.77)	0.77 (0.69–0.86)
Female gender	0.81 (0.76–0.86)	0.75 (0.69–0.82)	0.73 (0.67–0.80)	0.83 (0.62–1.11)	1.08 (0.98–1.19)	0.80 (0.73–0.87)
Diabetes duration	3.16 (2.97–3.38)	0.57 (0.51–0.63)	1.08 (0.97–1.20)	NA	NA	1.37 (1.25–1.51)
Hypertension	0.92 (0.86–0.99)	1.15 (1.05–1.25)	NA	NA	NA	NA
Dyslipidemia	NA	NA	NA	NA	NA	NA
Nephropathy	0.83 (0.72–0.96)	NA	NA	NA	NA	NA
Overweight	1.35 (1.27–1.45)	1.09 (0.99–1.18)	NA	NA	1.34 (1.21–1.49)	1.23 (1.12–1.34)
Cardiovascular comorbidity	NA	0.87 (0.79–0.96)	NA	NA	NA	NA
Peripheral vascular comorbidity	NA	0.84 (0.74–0.95)	NA	NA	NA	NA
Diabetes complications	1.14 (1.02–1.27)	0.87 (0.76–0.99)	0.83 (0.73–0.95)	NA	0.86 (0.74–1.00)	NA
Malignancy	0.87 (0.80–0.95)	0.87 (0.78–0.98)	0.81 (0.72–0.91)	0.77 (0.54–1.11)	NA	NA
Psychological disorder	0.81 (0.74–0.90)	NA	0.77 (0.67–0.88)	NA	0.61 (0.53–0.70)	0.81 (0.71–0.92)
≥5 chronic drugs	NA	0.61 (0.55–0.66)	NA	0.82 (0.60–1.10)	1.29 (1.15–1.44)	0.73 (0.67–0.80)
≥3 glucose-lowering drugs	NA	NA	1.39 (1.18–1.64)	NA	1.41 (1.17–1.70)	1.11 (0.97–1.29)
≥2 lipid-lowering drugs	1.53 (1.25–1.89)	1.31 (1.02–1.68)	NA	NA	NA	1.28 (0.98–1.63)
≥4 blood pressure-lowering drugs	1.36 (1.23–1.51)	NA	1.42 (1.25–1.61)	NA	NA	NA
Solo practice	NA	NA	0.86 (0.75–0.99)	NA	NA	NA
Assistant presence	0.72 (0.54–0.95)	NA	NA	1.54 (0.87–2.71)	NA	NA
Number of T2DM patients per practice	0.99 (0.99–0.99)	NA	0.99 (0.99–0.99)	NA	0.99 (0.99–0.99)	0.99 (0.99–0.99)

NA implies that characteristic was not included in the model since it was either an inclusion criterion (including p<0.2) or a part of the treatment measure

For glucose lowering drugs, patient and practice characteristics together reduced the practice level variance with 1.4%, and thereby explained 19.0% of the observed practice level variance in treatment (Table 3). Each of the tested patient characteristics explained less than 2% of the variance in the univariate analyses (S1 Appendix). Together, patient characteristics explained only 3.3% of the variance. Practice characteristics, in turn, explained 15.5% of the variance. For metformin, adjusting for patient characteristics reduced the practice level variance with 0.3% thereby explaining 7.5% of the variance in treatment. In the univariate analyses age of patient and use of ≥5 chronic drugs were the characteristics that explained the most of the practice level variance. Practice characteristics did not explain any practice level variance in treatment with metformin.

For lipid-lowering drugs and statins, patient and practice characteristics together reduced the practice level variance with 0.6% each, and thereby explained 20% and 6% of this variance respectively (Table 3). Age, treatment with 3 or more glucose-lowering drugs, and number of patients with T2DM per practice explained between 2.9% and 5.8% of the variance in treatment with lipid-lowering drugs in the univariate analyses (S1 Appendix). Together, patient characteristics explained 9.9% of the variance in treatment with lipid-lowering drugs and 0.3% of the variance in treatment with statins. Practice characteristics explained 8.3% and 6.0% of the variance respectively.

For blood pressure-lowering drugs and RAAS-blockers, patient and practice characteristics together reduced the practice level variance with 2.1% and 0.5% respectively, and thereby explained 9.9% and 13.4% of this variance (Table 3). A history of psychological comorbidity and number of T2DM patients per practice explained 3.7% and 4.3% of the variance in treatment with blood pressure-lowering drugs in the univariate analysis (S1 Appendix). Together, patient characteristics explained 6.2% of the variance in treatment with blood pressure-lowering drugs and 7.2% of the variance in treatment with RAAS-blockers. Practice characteristics explained 4.3% and 5.9% of the variance.

## Discussion

We observed considerable between practice differences in treatment with glucose-lowering, lipid-lowering, blood pressure-lowering drugs, and RAAS-blockers (IQR ranges of 10% or more) in T2DM patients. Smaller between practice differences in treatment were observed in treatment with metformin and statins (IQR ranges less than 8%). Not more than 10% of the observed differences, however, could be attributed to practice level, indicating that a significant part of the differences may be due to patient-level differences or random variance. Of these differences attributed to practice level, between 6% and 25% could be explained by the patient and practice level characteristics included in our study. Patient characteristics explained almost 10% of the differences in lipid-lowering treatment compared to less than 10% for the other treatments. Practice characteristics explained more than 15% of the differences in glucose-lowering treatment compared to less than 10% for the other treatments.

Several studies have described differences in treatment rates between general practices, showing sometimes wide ranges [27–31]. One study looked at between practice differences in treatment with glucose-lowering drugs in Danish patients, and found a two-fold difference in prescription rate between the 10 and 90 percentile [28] compared to a 1.4-fold difference in this study when calculated for these percentiles (data not shown). Another study looked at the differences in treatment with lipid-lowering and blood pressure-lowering drugs in the UK patients with diabetes and hypertension [27]. This study found the IQRs for treatment with lipid-lowering drugs of 11 and for blood pressure-lowering drugs of 8, which are slightly smaller in comparison to the IQRs of around 13 and almost 9 observed in our study. It thus appears that the practice variation we observed among Dutch general practices is lower for glucose-lowering drugs and similar for blood pressure-lowering drugs to that observed previously in other countries. The small between practice differences for treatment with metformin and statins may in part be due to restrictions on the reimbursement of some of the novel drugs, such as DPP-4 inhibitors, GLP-1 agonists and ezetimibe.

A recent review looking at patient characteristics associated with diabetes performance indicators did not find any consistent impact of demographics, complications, comorbidity, geography or care-seeking behavior [2]. They however included only studies addressing monitoring of risk factors, for which there may be few justified reasons not to conduct such monitoring. For prescribing treatment, we found that patient characteristics altogether explained at least 10% of the between practices differences for treatment with lipid-lowering drugs, but less than 10% for the treatment with blood pressure-lowering drugs and glucose-lowering drugs. For differences in treatment with lipid-lowering drugs but also with metformin, the patients’ age was relevant, implying that age of a patient influences the practitioners’ decisions to prescribe [32]. Since age-based prescribing in this case may be considered justified [33–35], this supports an age-stratified assessment of these treatment rates. For treatment with lipid-lowering drugs, the concomitant use of 3 or more glucose-regulating drugs explained 3% of the between practice variance. This suggests that there is a higher probability of receiving lipid-lowering drugs in patients with more severe diabetes. Since poor metabolic control is seen as an additional risk factor, starting statins is usually justified in such patients [24]. For metformin the concomitant use of 5 or more chronic drugs explained almost 7% of the variance. There seemed to be a shift from metformin to alternative treatment, including insulin, in patients with polypharmacy. This could be due to more complications and intolerability issues in these patients. On the other hand, the comorbidities and diabetes complications included in this study could not explain the between practice differences. These findings imply that the role of comedication and comorbidity in explaining between practice variance should be further investigated. Especially, more information is needed about other factors, such as disease severity, drug intolerance and medication adherence.

Practice characteristics explained at least 15% of the between practices differences for treatment with glucose-lowering drugs, and less than 10% for treatment with lipid-lowering and blood pressure-lowering drugs. Between practice differences in health care quality of patients with diabetes and the role of patient and practice characteristics in these differences were examined previously [36–38]. These studies, however, focused mainly on differences in monitoring of risk factors or differences in risk factor outcomes between practices. Similar to the findings of our study, they showed that the relatively small proportion (1–12%) of differences in quality of care can be attributed to differences between practitioners or practices [2, 36–38]. The number of T2DM patients per practice was the most relevant practice characteristic in our study, especially for receiving any treatment (yes/no). It explained between 5% and 13% of the treatment variance for glucose-lowering drugs, lipid-lowering drugs, blood pressure-lowering drugs, and RAAS-blockers. We observed a lower probability of being treated in practices with a higher number of T2DM patients. One explanation could be that these practices are more active in screening for diabetes, and therefore have more patients not yet in need of treatment [39, 40]. An alternative explanation is that the practice organization in large practices may be insufficient to provide optimal care. Although there is some evidence that practice size may negatively influence quality of care, this finding is not consistent [41]. Of the other practice characteristics, the presence of a physician’s assistant explained some additional variance in treatment with statins where it seemed that statins as a first-choice drug are more prescribed in practices with an assistant. This is in line with a finding in the UK, where prescribing of lipid lowering drugs was more guideline concordant in patients assessed by a project nurse [42]. Surprisingly, we observed a lower probability of being treated with glucose-lowering drugs in practices employing physician’s assistant. One explanation could be that the physicians’ assistants are more consistent with guidelines resulting in a higher number of newly diagnosed patients with T2DM per practice that may receive lifestyle advice first [40]. Overall, it appears that such general practice characteristics can only in part explain differences in treatment rates.

The primary care system organization in the Netherlands is comparable with other West-European countries [43]. The population of GIANTT cohort consists mainly of individuals of West-European origin and is comparable with the populations of type 2 diabetes patients from other regions of the Netherlands [3]. Around 12% of patients that were managed by a specialist for their diabetes were excluded from the analysis, since the primary aim of the study was to describe the differences in treatment quality of patients with diabetes between general practices. The study was based on data from the large general practice database containing a wide range of patient characteristics, treatment and comorbidity data. More than 90% of the differences in treatment between practices, however, cannot be explained at practice level. Part of this might be caused by the variance in other patient and practice level characteristics that we were not able to include in our study, e.g. intolerance or unwillingness of patients for taking specific or additional drug treatment, severity of disease, visit frequency or practice location [44]. Because of the cross-sectional design, we could not include the level of HbA1c, cholesterol, or blood pressure in the models. Instead we restricted all models to patients with an indication for treatment (e.g. diagnosis of hypertension or dyslipidemia). The comorbidity data in medical records are known to be incomplete [45], which would result in underestimating their influence on between practice variance. However, the comorbidity data in our study were enriched by manually coding text descriptions, resulting in higher comorbidity rates compared with that observed in a previous general practice study conducted in The Netherlands [46]. Moreover, practices with poor registration levels were excluded from our analysis. Finally, no data were available on practitioner level characteristics, such the physician’s knowledge or attitudes. Given the observed variance at practice level, there is a need to explore other, unmeasured practice or practitioner characteristics [44, 47].

Measuring the quality of treatment at practice level is part of various quality improvement initiatives [48–50]. In several countries external parties, such as insurance companies or professional organizations, use quality assessment to reward health care providers who meet predefined standards of quality. For fair assessment it is important to know whether the observed differences in quality of treatment between healthcare providers may be attributed to practice level, and to what extent they can be explained by differences in the underlying patient population. In such cases, either case-mix adjustment or stratification can be recommended [2]. The findings of our study imply that the between practice differences in tested treatment measures are only to a small degree affected by differences in the underlying patient population. Our study only supports to include age as a relevant patient characteristic to reduce justifiable differences in treatment rates, especially with lipid-lowering drugs. Other patient characteristics either do not explain the between practice difference in treatment or do not justify these differences. Of practice characteristics, we found that the number of T2DM patients per practice and presence of a physician’s assistant may explain between practice differences in treatment rates. This and also other modifiable practice characteristics should be identified and explored in future studies.

## Supporting Information

S1 Appendix Proportion of variance in treatment attributed to practice level with percentage reduction compared to empty model (%). (PDF)

S2 Appendix Description of the codelists and information required to run the algorithms. (PDF)
