# Supplementary material for: Role of Patient and Practice Characteristics in Variance of Treatment Quality in Type 2 Diabetes between General Practices
Source: PLoS One. 2016 Nov 2;11(11):e0166012. doi: 10.1371/journal.pone.0166012 (PMC5091743; doi:10.1371/journal.pone.0166012)
Supplement: S1 Appendix — (PDF) [file pone.0166012.s001.pdf]

S1 Appendix. Proportion of variance in treatment attributed to practice level with percentage reduction compared to empty model (%)

Model	Treatment with glucose-lowering drugs		Treatment with metformin		Treatment with lipid-lowering drugs		Treatment with statins		Treatment with blood pressure-lowering drugs		Treatment with RAAS-blockers	
	Variance at practice level	% explained variance	Variance at practice level	% explained variance	Variance at practice level	% explained variance	Variance at practice level	% explained variance	Variance at practice level	% explained variance	Variance at practice level	% explained variance
Empty	7.53		3.60		3.13		10.26		8.55		3.89	
Age included	7.56	-0.40	3.39	5.83	3.01	3.83	10.2	0.58	8.53	0.23	3.85	1.03
Gender included	7.54	-0.13	3.54	1.67	3.08	1.60	10.06	1.95	8.55	0.00	3.79	2.57
Diabetes duration included	7.48	0.66	3.52	2.22	3.13	0.00	10.26	0.00	8.57	-0.23	3.83	1.54
Hypertension included	7.52	0.13	3.60	0.00	3.14	-0.32	10.27	-0.10	NA	NA	NA	NA
Dyslipidemia included	7.53	0.00	3.60	0.00	NA	NA	NA	NA	8.61	-0.70	3.91	-0.51
Nephropathy included	7.52	0.13	NA	NA	NA	NA	NA	NA	NA	NA	NA	NA
Overweight included	7.51	0.27	3.64	-1.11	3.15	-0.64	10.24	0.19	8.63	-0.94	3.96	-1.80
Cardiovascular comorbidity included	7.61	-1.06	3.67	-1.94	NA	NA	NA	NA	NA	NA	NA	NA
Peripheral vascular comorbidity included	7.53	0.00	3.65	-1.39	NA	NA	NA	NA	NA	NA	NA	NA
Diabetes complication included	7.64	-1.46	3.64	-1.11	3.11	0.64	10.12	1.36	8.48	0.82	3.90	-0.26
Malignancy included	7.48	0.66	3.66	-1.67	3.09	1.28	10.48	-2.14	8.48	0.82	3.88	0.26
Psychological disorder included	7.45	1.06	3.61	-0.28	3.11	0.64	10.25	0.10	8.23	3.74	3.85	1.03
≥5 chronic drugs included	7.54	-0.13	3.36	6.67	3.16	-0.96	10.18	0.78	8.49	0.70	3.90	-0.26
≥3 glucose-lowering drugs included	NA	NA	NA	NA	3.04	2.88	10.25	0.10	8.42	1.52	3.89	0.00
≥2 lipid-lowering drugs included	7.50	0.40	3.61	-0.28	NA	NA	NA	NA	8.52	0.35	3.86	0.77
≥4 blood pressure-lowering drugs included	7.48	0.66	3.58	0.56	3.10	0.96	10.25	0.10	NA	NA	NA	NA
Solo practice	7.31	2.92	3.60	0.00	3.12	0.32	10.03	2.24	8.56	-0.12	3.88	0.26
Assistant presence	7.25	3.72	3.60	0.00	3.13	0.00	9.64	6.04	8.55	0.00	3.82	1.80
Number of T2DM patients per practice	6.54	13.15	3.62	-0.56	2.95	5.75	10.28	-0.19	8.18	4.33	3.66	5.91
Model 1: patient characteristics	7.28	3.32	3.33	7.50	2.82	9.90	10.23	0.29	8.02	6.20	3.61	7.20
Model 2: practice characteristics	6.36	15.54	3.62	-0.56	2.87	8.31	9.64	6.04	8.18	4.33	3.66	5.91
Model 3: patient and practice	6.10	18.99	3.33	7.50	2.50	20.13	9.64	6.04	7.70	9.94	3.37	13.37

NA implies that characteristic was not included in the model since it was either an inclusion criterion for or part of the treatment measure
