# Supplementary material for: Role of Patient and Practice Characteristics in Variance of Treatment Quality in Type 2 Diabetes between General Practices
Source: PLoS One. 2016 Nov 2;11(11):e0166012. doi: 10.1371/journal.pone.0166012 (PMC5091743; doi:10.1371/journal.pone.0166012)
Supplement: S2 Appendix — (PDF) [file pone.0166012.s002.pdf]

S2 Appendix. Description of the codelists and information required to run the algorithms

gp double	%10.0g	General practice GIANTT code
patient double	%10.0g	Patient identifier at general practice
solo_gp double	%1.0g	Solo GP practies (yes/no)
assistant double	%1.0g	Presence of practice assistant (yes/no)
female double	%1.0g	Female gender (yes/no)
nephropathy double	%1.0g	History of nephropathy diagnosis according to ICPC-1 classification (yes/no)
polypharmacy_~c double	%1.0g	Use of 5 or more chronic drugs (yes/no)
polypharmacy~gl double	%1.0g	Use of 3 or more drug from different glucose-lowering drug class (yes/no)
polypharmacy~ll double	%1.0g	Use of 2 or more drug from different lipid-lowering drug class (yes/no)
polypharmacy_~h double	%1.0g	Use of 4 or more drug from different glucose-lowering drug class (yes/no)
hypertension double	%1.0g	History of hypertension diagnosis according to ICPC-1 classification (yes/no)
dyslipidemia double	%1.0g	History of dyslipidemia diagnosis according to ICPC-1 classification (yes/no)
overweight double	%1.0g	Overweigth diagnosis according to ICPC-1 classification (yes/no)
antidm_2012_cat double	%1.0g	Use of glucose-lowering treatment in the last 4 months of 2012
metformin_201~t double	%1.0g	Use of metformin in the last 4 months of 2012
antilip_2012_~t double	%1.0g	Use of lipid-lowering treatment in the last 4 months of 2012
statins_2012_~t double	%1.0g	Use of statins in the last 4 months of 2012
antihyp_2012_~t double	%1.0g	Use of blood pressure-lowering treatment in the last 4 months of 2012
ras_2012_cat double	%1.0g	Use of ACE-i or ARB in the last 4 months of 2012
cvd_cat1 double	%1.0g	History of cardiovascular diagnoses according to ICPC-1 classification (yes/no)
pvd_cat1 double	%1.0g	History of perophetal vascular diagnosis according to ICPC-1 classification (yes
diabcl_cat1 double	%1.0g	History of diabetes complications according to ICPC-1 classification (yes/no)
malig_cat1 double	%1.0g	History of malignancy diagnosis according to ICPC-1 classification (yes/no)
psy_cat1 double	%1.0g	History of psychological diagnosis according to ICPC-1 classification (yes/no)
age_70 double	%1.0g	Age >= 70 (yes/no)

age_80 double	%1.0g	Age >= 80 (yes/no)
duration_over2 double	%1.0g	Diabetes duration >= 2 years (yes/no)
n_pat_gp float	%9.0g	Number of type 2 diabetes patient per practice (numeric)
Sorted by: gp patient		
